# Aids to Nursing

**Published:** 1888-04-28

**Authors:** M. M. Basil

**Affiliations:** Author of "The Commoner Accidents to Life and Limb.


### Aids to Nursing.

By M. M. Basil, M.A., M.B., C.M., Author of "The Commoner Accidents to Life and Limb."

*(Continued from p. 6.)*

### Bathing and Baths.

A bath should be so constructed that the hot and cold water can be regulated independently of each other. It is best to have them fall into the bath from a higher level than the overflow-pipe: not from a rose at the bottom of the bath. The filling and emptying of the bath should not take much time. No nurse who has to attend to the bathing of several patients can stand the interminable trickling from the supply or waste pipe of the baths which are fitted up by some plumbers. The patience of attendants on the sick must be carefully preserved, as it is a valuable remedial store against numberless calls made on it during disease.

The safest course in getting a bath ready is to add hot water to a supply of cold. If the nurse is extremely careful the reverse arrangement may be adopted. It has the advantage of raising the general temperature of the room, but it is a very fruitful source of disagreeable accidents. Always test the temperature of the water first with a thermometer and then with your arm. In rare cases, in the absence of a thermometer, the bare elbow may be used for ascertaining the temperature of the water. But, as a rule, the hand of a person in good health is no standard of the degree of heat that can be comfortably borne by the sick. The general rule is that most diseases make the skin oversensitive to heat and cold. You must not quite ignore the patient's sensations because to you the water feels just com fortable. The same patient may differ very considerably from hour to hour in the opinion he expresses as to the temperature of a bath of the same thermometic reading. The general health of the body, especially the nervous tone; the amount of nourishment taken; the soothing influence of a few hours' sleep or the reverse condition, are some of the important modifying factors which determine the effect produced by any given temperature on the skin. Within certain limits, there is nothing hot or cold but thinking makes it so.

Then, again, it is quite possible that without your knowing it the sensory function of your hand may be in an abnormal state. To realise the possibility of this condition, you have only to think of the cases of nervous disease that come under observation during your training. You will frequently see patients who are perfectly paralysed so far as movement of their limbs is concerned, while sensation in the parts is quite normal. You will also sometimes see the reverse condition, viz., that the limb can be moved, but either its ordinary feeling is lost or diminished, or sensation to heat and cold alone is lost. You can therefore never be sure that your hand alone is a reliable measure of the degree of heat safe for your patient.

To impress on you the importance of this point, I shall briefly describe an experience full of lessons to us all. Several years ago a peculiar epidemic broke out in Elbing among new-born babies. For some time it was looked upon as an epidemic of infantile lock-jaw, or *trismus neonatorum*. Careful investigation showed that the epidemic spread in a most zigzag and unphilosophical manner. No curve or any conceivable modification of it could represent it, or give the least clue as to its law of incidence. It was, however, observed to be a close follower of the erratic movements of a particular midwife. It was then found out that this German representative of the house of Gamp, Prig, and Co. was perfectly unable to make out the least difference between water at 28° C. and 33° C.—*i.e.*, nearly 82° F. and 91° F. The epidemic of lock-jaw was thus merely a general boiling of infants.

What is infinitely more frequent, your patient may have paralysis of sensation which has not been suspected in the least, and you may find him scalded very severely without giving you any warning of the accident.

To know the temperature of the bath correctly, you must stir up the water so as to mix the hot and cold layers of it, then plunge in the thermometer, move it about for some minutes, and draw it up vertically to determine its reading. The process must not be converted into a mere formality. The momentary plunge of a thermometer into one portion of water is worse than useless, since in place of remaining in blissful ignorance on the subject, you are deceived with assurances of false safety. There is no witchcraft in a thermometer. To make it serviceable you must obey the laws regulating its use.

If the bath is so constructed that the hot water supply comes from a rose at the bottom of it, you must, immediately before putting the patient into it, ascertain the temperature of the water with your hand, to be sure that no boiling water has entered it after the thermometric reading. You may otherwise scald your patient very severely if the regulator for turning off the hot water is out of order. Certainly it is no fault of the nurse if the plumber's work has been done badly, but should the patient be scalded it is but small comfort to be assured that it was no blame of yours. Long after "Martin Chuzzlewit" had been written, and every biographer of Dickens had assured the world that his genius had made bad nursing an impossibility, a patient was scalded to death by two nurses. It would appear that the construction of the bath was to blame in that case.

One may remark in passing that though it is scarcely possible that Mrs. Gamp or her prototype existed as a nurse within recent times, still it would be advisable for everyone connected with the care of the sick to make a study of her life. No one acquainted with hospital work would be willing to believe that any of the London institutions had the slightest credit for her training. If the picture, however, was drawn from a study of some of the irregular or so-called self-trained midwives, then Dickens has not yet succeeded in finishing off the race.

The bath should be of about the same temperature from first to last. This is true of all baths, but much more so of the partial baths, such as the arm or the leg bath. When water is to be added subsequently to keep up the temperature of the bath, you must guard against scalds to your patient. Under no conditions should a second patient be placed in the water which has been already used. There should be no bits of detached paint, sand, cinders, or pieces of slate in the bath. As most people become ridiculously nervous in disease, you must never get tired of assuring and reassuring your patient that no harm is going to be done during the bathing. The towel must be warmed uniformly, so that no portion of it is too hot for an over-sensitive skin. The head of the patient should in no case be submerged under the water, and if there is any difficulty of breathing even the chest should not be submerged. The pressure of the water on the chest walls in such cases interferes much with the process of respiration, which is already carried on with difficulty.

The temperature of the water, and the time during which the patient must be kept in it, are important factors in determining the effects produced by every bath. You should therefore in all cases of doubt distinctly ascertain each of these points from your superiors. Without very definite instructions you should not, as a rule, undertake to give a bath if it is to be over 100° F., or below 60° F.; 50° F. is very cold. Take especial care as to the temperature in cases of children, particularly of babies.

With reference to their temperature, baths are spoken of as cold, cool, temperate, tepid, warm, and hot. Of these, you should only remember that the range of the tepid one is from 85° to 92°, the warm one from 92° to 98°, and the hot one from 98° to 105° F.

(*To be continued.*)

